# A More Targeted and Selective Use of Implantable Loop Recorders Improves the Effectiveness of Syncope Units: A Single-Center Experience

**DOI:** 10.3390/life14070871

**Published:** 2024-07-12

**Authors:** Stefanos Archontakis, Evangelos Oikonomou, Konstantinos Sideris, Panagiotis Dourvas, Nikias Milaras, Panagiotis Kostakis, Tzonatan Klogkeri, Epameinondas Triantafyllou, Panagiotis Theofilis, Ioannis Ntalakouras, Petros Arsenos, Athanasia Gkika, Konstantinos Gatzoulis, Skevos Sideris, Dimitris Tousoulis

**Affiliations:** 1 Department of Cardiology, Hippokration General Hospital, 114 Vasilisis Sofias Str., 11527 Athens, Greece; stef6arch@yahoo.com (S.A.); kostassid@med.uoa.gr (K.S.); panos_kost@hotmail.com (P.K.); tzonklog@med.uoa.gr (T.K.); nodastrianta@med.uoa.gr (E.T.); ioantalak@gmail.com (I.N.); skevos1@otenet.gr (S.S.); 2 Third Cardiology Division, Medical School, University of Athens, Sotiria Thoracic Diseases Hospital, 152 Mesogeion Ave., 11527 Athens, Greece; boikono@med.uoa.gr; 3 First Cardiology Division, Medical School, University of Athens, Hippokration General Hospital, 114 Vasilisis Sofias Str., 11527 Athens, Greece; ptheofilis@med.uoa.gr (P.T.); arspetr@otenet.gr (P.A.); kgatzoul@med.uoa.gr (K.G.); 4 Department of Cardiac Surgery, Hippokration General Hospital, 114 Vasilisis Sofias Str., 11527 Athens, Greece

**Keywords:** syncope unit, implantable loop recorder, unexplained syncope, tilt table test

## Abstract

Purpose: Syncope remains a common medical problem. Recently, the role of dedicated syncope units and implantable loop recorders has emerged in the investigation of unexplained syncope. This study aims to investigate the possibilities for a more rational and targeted use of various diagnostic tools. Methods: In this retrospective single-center study, 196 patients with unexplained syncope were included between March 2019 and February 2023. Various diagnostic tools were utilized during the investigation, according to clinical judgement. Patients were retrospectively allocated into Group A (including those who, among other tests, underwent loop recorder insertion) and Group B (including patients investigated without loop recorder implantation). Data were compared with Group C, including patients assessed prior to syncope unit establishment. Results: There was no difference between Group A (*n* = 133) and Group B (*n* = 63) in the diagnostic yield (74% vs. 76%, *p* = 0.22). There were significant differences between Groups A and B regarding age (67.3 ± 16.9 years vs. 48.3 ± 19.1 years, *p* < 0.001) and cause of syncope (cardiogenic in 69% of Group A, reflex syncope in 77% of Group B, *p* < 0.001). Electrocardiography-based diagnosis occurred in 55% and 19% of Groups A and B, respectively (*p* < 0.001). The time to diagnosis was 4.2 ± 2.7 months in Group A and 7.5 ± 5.6 months in Group B (*p* < 0.001). In Group C, the diagnostic yield was 57.9% and the electrocardiography-based diagnostic yield was 18.3%. Conclusions: A selective use of loop recorders according to clinical and electrocardiographic characteristics increases the effectiveness of the structured syncope unit approach and further preserves financial resources.

## 1. Introduction

Syncope remains a common medical problem with significant social consequences and healthcare costs [1,2,3]. Numerous studies provide convincing evidence with regard to the value of developing structured syncope facilities (i.e., syncope units), compared to conventional practice, in reducing syncope’s underdiagnosis and misdiagnosis, improving the diagnostic yield, reducing the hospitalization rate and the number of unnecessary diagnostic tests performed, and reducing the overall cost per diagnosis [4,5,6,7,8,9]. Consequently, the formation of dedicated syncope units with specialized staff and access to appropriate diagnostic options and therapies is currently recommended, in order to provide a standardized approach for the diagnosis and management of syncope [4,10].

The combination of history, clinical evaluation, and 12-lead electrocardiography (ECG) remains the cornerstone for achieving a diagnosis. However, early use of an implantable loop recorder (ILR) further improves diagnostic yield and was proven to be more cost-effective compared to other strategies in patients with unexplained syncope, especially when an arrhythmic etiology is suspected [10,11,12,13]. In these patients, long-term ECG monitoring allows for an accurate correlation between symptoms and electrocardiographic findings. Therefore, the current guidelines favor ILR implantation early in the investigation of recurrent unexplained syncope [10], whereas remote ILR monitoring appears to further enhance the diagnostic power and to reduce the time to diagnosis and the risk of syncope recurrence [14,15].

Despite these improvements, diagnostic investigation of unexplained syncope remains challenging and costly. Moreover, the use of loop recorders, albeit cost-effective, may have a significant financial impact for healthcare systems when used indistinctively, which, in turn, may lead to their underutilization due to the lack of financial resources.

The aim of this study is to compare the diagnostic yield of the different evaluation strategies for syncope and to provide additional information regarding a more rational and targeted use of various diagnostic tools.

## 2. Methods

### 2.1. Study Design and Patient Population

This retrospective, open-label study was performed in a single syncope unit in Greece. We included all patients with unexplained syncope admitted to our facility between March 2019 and February 2023.

The syncope unit was established on March 2019 and is based at the Cardiology Department of Hippokration General Hospital, Athens, Greece. It operates mostly as an outpatient structure, as well as by reviewing patients admitted through the Emergency Department, according to the current guidelines [4,10]. Outpatient referrals for further assessment and consulting originate either from the various outpatient departments of our hospital or other hospitals, or from the Emergency Department; however, self-referral is also offered.

The inclusion criteria for participation in this study were as follows: (a) patient suffering from episodes fulfilling the definition of pre-syncope or syncope (i.e., transient loss of consciousness (TLOC) due to cerebral hypoperfusion, characterized by a rapid onset, short duration, and spontaneous complete recovery), (b) patient complaining of episodes fulfilling the definition of TLOC (i.e., state of real or apparent LOC with loss of awareness, characterized by amnesia for the period of unconsciousness, abnormal motor control, loss of responsiveness, and a short duration), (c) patients referred from other facilities for episodes characterized as ‘unexplained syncope’ (US) or unexplained ‘falls’, and (d) absence of any other life-threatening comorbidity or systematic disease that could potentially limit the prognosis to <1 year [10].

Individuals were reassessed according to the ‘initial evaluation’ scheme in the syncope unit, by obtaining a detailed history of the episode, reviewing medical and drug history, performing a clinical examination in which we included an active standing test and carotid sinus massage when clinically relevant and, finally, performing a 12-lead electrocardiogram. Data were recorded in hard-copy forms as well as in stored electronic forms. The patients’ demographics were systematically recorded. 

Further assessment strategy was individualized according to clinical judgement and evidence based on the ESC guidelines. Patient preference was also considered. All patients underwent a 24 h ECG ambulatory monitoring, along with basic hematological and biochemical blood tests including a full blood count, renal function tests, and electrolyte and liver function tests. In addition, all patients underwent a transthoracic echocardiogram. Selection of the ‘ILR pathway’ was based on the current scoring system proposed by the ESC as well as the clinical experience of the physicians involved in the patient investigation. Clinical characteristics suggesting a high probability of arrhythmic mechanisms, such as the presence of palpitations, fatigue, lack of triggers or prodromes, symptoms in the supine position, etc., in combination with the presence of ECG abnormalities, guided our decision for selecting the most adequate test for further investigating syncope.

Additional tests were performed in a case-by-case strategy and included a possible tilt table test (TTT), electrophysiological study (EPS), and/or implantation of a loop recorder. Patients were stratified according to the scheme proposed by the ESC. Close follow-up, TTT, and non-invasive ECG repeat recording were preferred in cases where an autonomic mechanism was suspected, whereas in patients with a suspected cardiogenic syncope or cardioinhibitory mechanism, ILR implantation was proposed. For the TTT, the standard 20 min Italian Protocol was performed, followed by a provocation phase during which 400 micrograms of sublingual nitrogen triglyceride spray was administrated. A functional test (e.g., treadmill stress test, stress echo, or SPECT) to assess the presence of myocardial ischemia and/or to investigate exercise-induced or post-exercise-induced syncope was also considered when clinically relevant. Moreover, coronary angiography was performed when the possibility of ischemia was high. When required, patients were referred to the internal medicine or neurology outpatient department for assessment.

All patients undergoing implantation of an implantable loop recorder received a Reveal LINK^TM^ or a LINK II ^TM^ Insertable Cardiac Monitoring System (Medtronic Inc., Minneapolis, MN, USA), and in all cases remote monitoring (CARELINK^TM^ NETWORK, Medtronic Inc., Minneapolis, MN, USA) was used for follow-up. The ILR recordings were reviewed daily. Spontaneously recorded arrhythmias were classified according to the ISSUE study criteria as follows: Type 1: Asystole (1A: sinus arrest, 1B: sinus bradycardia plus atrioventricular block, 1C: atrioventricular block); Type 2: Bradycardia; Type 3: No or slight rhythm variations; Type 4: Tachycardia. Patient-triggered events, recorded as ‘symptoms’, were also assessed and, in addition, the patient was contacted to assess the nature of the symptom. Patients were also advised to contact the center in the presence of unexplained symptoms. ECG diagnosis was considered when a combination of arrhythmias and relevant symptoms was present, or in the presence of serious arrhythmias, independent of the duration of monitoring or the frequency of events.

Follow-up visits at the syncope unit were scheduled on an individual basis. At each visit, the clinical status and history were recorded, and the strategy of investigation was revised accordingly. Unscheduled visits were also possible according to symptoms. Moreover, urgent or elective hospital admissions were decided on a case-by-case basis according to the clinical findings. All patients were followed up for at least 12 months after their initial assessment.

The primary endpoint of this study was a definite diagnosis of syncope. Secondary endpoints were (a) obtaining an ECG diagnosis and (b) the time from initial presentation in our facility to final diagnosis.

In the present study, patients were retrospectively allocated to two study groups: Group A, including those who were assessed in the syncope unit and underwent ILR insertion, and Group B, including those who were investigated in the syncope unit without ILR implantation. In addition, data were collected from a historical cohort of adult patients who were assessed in the Outpatient Department of our hospital prior to the syncope unit’s establishment, between September 2016 and February 2019 (Group C). 

### 2.2. Statistical Analysis

Data are presented as absolute numbers and percentages if categorical, and as means ± standard deviations if continuous. All variables were tested for normality of distribution with p = p plots and the Kolmogorov–Smirnov test. For categorical variables, differences between different studied groups were tested with the chi-squared test or Fisher’s exact test as appropriate, and for continuous variables they were tested with analysis of variance (ANOVA). Intergroup differences were evaluated after correction for multiple comparisons. The *t*-test was used to evaluate differences between normally distributed continuous variables between two studied categories. Statistical calculations were performed using SPSS software (version 27.0; SPSS Inc., Chicago, IL, USA) and GraphPad Prism (version 8, GraphPad, Boston, MA, USA).

## 3. Results

During the 36-month period (1 March 2019 to 28 February 2023), a total of 196 patients were referred to our syncope unit (5.4 admissions/month). The mean age of this population was 61.2 ± 19.9 years (range: 15–92 years old), and 97 patients (49.5%) were female. Referrals were from the Emergency Department in 17 cases (8.7%), from the Outpatient Department of our hospital in 60 cases (30.6%), and from other hospitals and primary care facilities in 111 cases (56.6%), whereas self-referrals represented 8 cases (4.1%).

Retrospective research was also conducted in the patient records of the Outpatient Cardiology Department during a 30-month period prior to the syncope unit’s establishment. During that period, 71 patients visited the outpatient clinic, 37 of whom were female (52.1%). The mean age of this population was 57.8 ± 16.4 years.

As mentioned, patients assessed in the syncope unit were retrospectively allocated to Group A (implantation of an ILR, among other tests) and Group B (investigated in the syncope unit without ILR implantation). These data were compared with data from a historical cohort assessed in the Outpatient Cardiology Department (Group C). None of the patients of Group C received an ILR. Patient demographics, clinical characteristics, and investigational strategies are presented in Table 1 and Table 2. There were no ILR implant-related complications. No hardware-related adverse events were recorded during the study period. In addition, no adverse events related to the recorded arrhythmias were reported.

The mean age of the patients in Group A (*n* = 133), Group B (*n* = 63), and Group C (*n* = 71) was 67.3 ± 16.9 years, 48.3 ± 19.1 years, and 57.8 ± 16.4 years, respectively. There were no differences regarding female sex between Groups A, B, and C (49.6% vs. 49.2% vs. 52.1%, *p* = 0.93).

Active standing revealed an abnormal pattern in three patients in Group A, and in another three patients of Group B. The test was considered to be clinically relevant and pathognomonic for orthostatic hypotension in all three patients of Group B but only in one patient of Group A. In the other two patients of Group A, history was not consistent with orthostatic hypotension. On the other hand, carotid sinus massage (CSM) revealed carotid sinus hypersensitivity with a pause > 5 s in 2 of the 32 patients of Group A. CSM was normal in all eight cases of Group B.

In patients of Group A, ambulatory 24 h ECG monitoring was insignificant in 51 cases (38.5%) and had minor (albeit not pathognomonic) findings in 82 patients, such as low mean heart rate, significant number of premature atrial or ventricular beats, and runs of supraventricular or non-sustained ventricular tachycardia. In the 18 patients of this group, who underwent inpatient monitoring for 24 h, no significant arrhythmic events were recorded. In the patients of Group B, ambulatory 24 h ECG monitoring was without findings in 39 cases (61.9%), revealed significant bradycardia in 1 case, and showed minor arrhythmic events in 23 cases.

Echocardiography was performed in all cases, either in our laboratory or in other medical facilities, as an attempt to assess the structural condition of the heart. In none of the 196 patients was echocardiography diagnostic for syncope in our cohort; however, it played a pivotal role in selecting the subsequent diagnostic steps.

The tilt table test was performed in 10.5% and 79.4% of patients from Groups A and B, respectively (*p* < 0.001). In the 20 of the 50 patients of Group B, where the TTT was performed, the test was diagnostic, showing a cardioinhibitory reaction in six cases, a mixed cardioinhibitory/vasodepressive reaction in nine cases, and a vasodepressive reaction in five cases with reproduction of clinical symptoms. In the 4 of the 14 patients of Group A, the test was diagnostic, showing a mixed cardioinhibitory/vasodepressive reaction in one case and a vasodepressive reaction in three cases with reproduction of clinical symptoms. Electrophysiological study was performed for 20 patients in Group A and in 4 patients in Group B. The study was considered to be suggestive of atrioventricular conduction disorders in cases of an HV interval > 70 ms. In addition, a prolonged corrected sinus node recovery time (cSNRT) and an early-occurring atrioventricular block during rapid atrial pacing were also recorded. All 24 patients of Groups A and B underwent programmed ventricular stimulation with a standard protocol including two and three extra stimuli in sequential fashion following an eight-beat drive train of 550 ms cycle length that was repeated using a drive train of 400 ms cycle length. In all 20 patients of Group A, the results were within the normal limits. In two of the four patients of Group B, with ischemic cardiomyopathy and mildly reduced ejection fraction, ventricular tachycardia was induced during programmed ventricular stimulation.

In Group A, most of the participants had an ILR-based diagnosis. In 5 cases, a cardioinhibitory reflex response was recorded, whereas in 67 cases the cause was arrhythmic. In the later subgroup, 17 patients were recorded with tachycardia (6 with atrial fibrillation, 8 with supraventricular tachycardia, and 3 with ventricular tachycardia) and 50 with bradycardia (29 with conduction disorders and 21 with sinus arrest).

### 3.1. Primary Endpoint: Diagnostic Yield of Different Strategies

**Figure 1:**
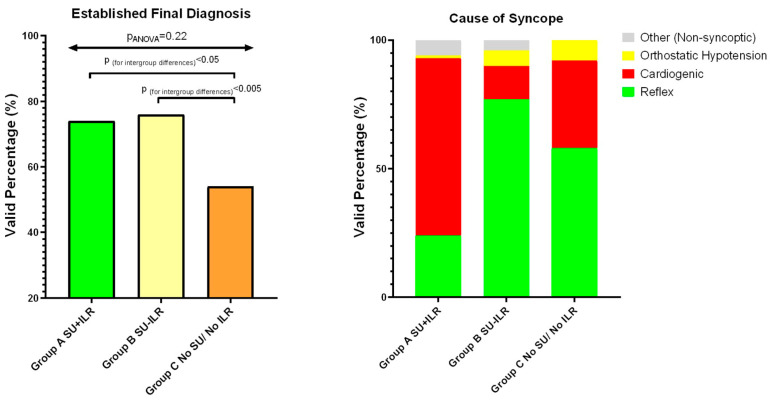
Left panel: diagnostic yield in Groups A, B, and C, calculated at 73.7%, 76.2%, and 53.5%, respectively. Right panel: Causes of syncope in patients with an established final diagnosis in Groups A, B, and C.

The patients were followed up for a minimum of 12 months (range: 12–48 months), whereas the total follow-up time (25.6 ± 10.6 months for Group A vs. 28.1 ± 12.4 months for Group B, *p* = 0.15) did not differ between the two studied groups. A certain final diagnosis was established in 98 of the 133 patients (73.7%) in the ILR/SU group and in 48 of the 63 patients (76.2%) in the group assessed in the SU with no utilization of an ILR (*p* = 0.22), demonstrating a similar diagnostic performance between the two groups. The diagnostic yield was superior compared to that of the historic conventional (non-SU/non-ILR) group (53.5%) (Table 3, Figure 1). In Group A, most of the cases were due to cardiogenic syncope (69.4%), whereas in Group B the dominant cause was reflex syncope (77.1%) (Table 3, Figure 1). 

### 3.2. Secondary Endpoints

**Figure 2:**
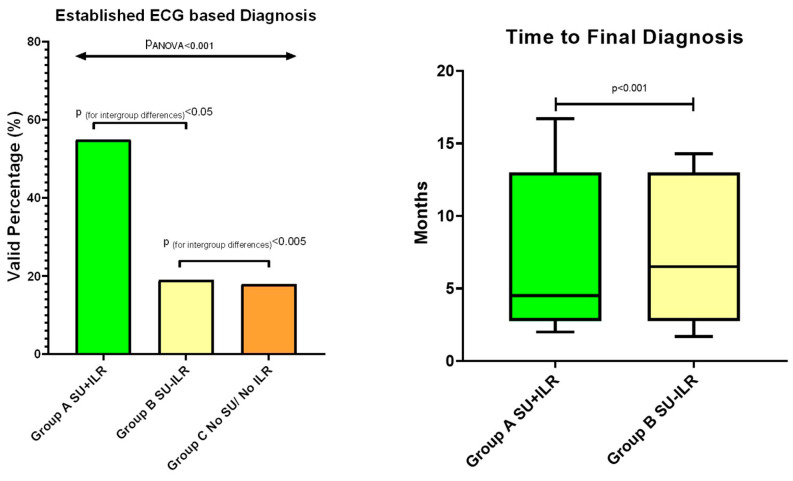
Left panel: Established electrocardiographic-based diagnosis in Groups A, B, and C, calculated at 55.1%, 19%, and 18.3%, respectively. Right panel: Mean time to final diagnosis in Groups A and B, calculated at 4.2 ± 2.7 months and 7.5 ± 5.6 months, respectively.

Cardiogenic syncope in Group A was mostly due to bradycardia and/or pauses due to either sick sinus syndrome or atrioventricular block. Sixty-seven of the sixty-eight patients diagnosed with cardiogenic syncope in this group had an ECG-based diagnosis made with the implanted ILR. In addition, in 5 of the 23 patients with reflex syncope in Group A, a cardioinhibitory event was recorded with the ILR, confirming the diagnosis. Overall, in Group A, an ECG-based diagnosis was achieved in 72 of the 133 patients (55.1%). On the other hand, in Group B, final diagnosis was based on the ECG in 12 cases (19%), i.e., in 6 patients with a combination of a cardioinhibitory reaction and reproduction of symptoms during the tilt table test, and in 6 patients with cardiogenic syncope. In the later subgroup, ventricular tachycardia was induced during EPS in two cases, whereas during follow-up, symptomatic bradycardia was revealed in three cases and symptomatic atrial tachycardia in one patient on 12-lead ECG or follow-up 24 h ECG monitoring. Finally, in the conventionally treated group, an ECG-based diagnosis was established in 13 of the 71 patients (18.3%) (Table 3, Figure 2).

Final diagnosis in Group A was achieved within a mean of 4.2 months after the initial assessment at the syncope unit, compared to 7.5 months in Group B (*p* < 0.001) (Table 3, Figure 2).

### 3.3. Therapy

In Group A, a pacemaker was implanted in 47 cases, a lead correction was performed in 1 case, and two patients refused pacemaker implantation. Tachycardia was recorded in 17 cases in the form of atrial fibrillation with rapid ventricular response (*n* = 6) that was treated medically, supraventricular tachycardia (*n* = 8) treated with ablation in 7 cases and atrioventricular junction ablation in one case (in a patient who already had an implantable cardioverter defibrillator), or ventricular tachycardia (*n* = 3) treated with ICD implantation in 1 case and conservatively in 2 cases. Twenty-three patients were diagnosed with reflex syncope, one of whom was treated with leadless pacemaker implantation, while the rest were treated conservatively. In Group B, one patient with cardiogenic syncope received an implantable cardioverter defibrillator (ICD), another received a biventricular pacemaker/defibrillator, and three received a pacemaker. One patient was diagnosed with supraventricular atrial tachycardia and was treated medically. Thirty-six of the thirty-seven patients diagnosed with reflex syncope were treated conservatively, whereas one patient received a pacemaker. In Group C, all 22 patients with reflex syncope were treated conservatively. Seven patients with symptomatic bradycardia/pauses received a pacemaker; one received an ICD and three underwent ablation for supraventricular tachycardia. Two patients were diagnosed with fast paroxysmal atrial fibrillation and were treated medically. All patients with orthostatic hypotension were treated according to the current ESC-guidelines with conservative measures, without using drug therapy. Patients with non-syncopal causes were treated accordingly. 

## 4. Discussion

Syncope remains a challenging clinical presentation, due to the inhomogeneity of its causes, including a large spectrum of both benign and potentially life-threatening conditions. Furthermore, even when the cause is benign, studies have revealed a significant adverse socioeconomic impact for both individuals and healthcare systems [2,3,16]. The combination of clinical presentation and 12-lead ECG remains the cornerstone for diagnosis; however, the data are often ambiguous. There is growing evidence demonstrating the pivotal role of dedicated syncope units and the clinical utility of ILRs in increasing the diagnostic yield [1,2,3,4,5,6,7,8,9,10,11,12,14]. Thus, current recommendations favor the use of ILRs in the early stage of the diagnostic investigation [10,17]. However, since the cost of diagnosis remains high, in clinical practice there are still restrictions on the utilization of ILRs in most of the patients with unexplained syncope.

In the present study, in 196 patients assessed in the syncope unit, we decided to implant a loop recorder when the likelihood of cardiogenic syncope was higher based on clinical, demographic, and electrocardiographic criteria, whereas in those with a higher probability of reflex syncope we insisted on the use of other diagnostic tools, such as the TTT and repeated 24 h ECG monitoring. We demonstrated a similar diagnostic yield between these two groups, since a final diagnosis was achieved in 73.7% and 76.2%, respectively. In the former group of patients, in whom an ILR was implanted, the mean age was 67.3 years, the final diagnosis was cardiogenic syncope in 69.4%, and the diagnosis was based mostly on electrocardiographic findings, since ECG-based diagnosis was calculated at 55.1%. On the other hand, in the latter group, the mean age was significantly lower, at 48.3 years, most of the patients were diagnosed with reflex syncope (77.1%), and ECG-based diagnosis was only 19%. Despite these different characteristics, the diagnostic yields were comparable between the groups. Based on these findings, we may assume that a more selective use of the ILR in patients according to their clinical and electrocardiographic characteristics may be an acceptable diagnostic pathway. An echocardiogram, initial 24 h electrocardiographic ambulatory monitoring, and blood tests could be added to the initial assessment to further assess the patients’ symptoms. In cases of suspected reflex syncope, a tilt table test may be used as a first-line test, whereas in cases of probable cardiogenic syncope, ECG recording with ILR implantation may be preferable. According to our data, this strategy appears to be associated with a satisfactory diagnostic yield, while also minimizing the cost of investigation. Despite the fact that several studies have questioned the role of EPS, other authors have shown that abnormal ECG findings on non-invasive testing are well correlated with potential brady- or/and tachyarrhythmic causes of syncope in EPS of patients with undiagnosed syncope [18,19]. We believe that the electrophysiological study is a useful diagnostic tool and could further assist the investigational process in selected cases.

An important finding is that diagnostic yields may be similar between the two groups; however, the time to diagnosis was significantly reduced in the group of patients in whom an ILR was implanted. Diagnosis was achieved within a mean of 4.2 months in these patients, compared to 7.5 months in patients without ILRs.

Another finding from our work is that, similar to previous studies, our results support the formation of dedicated syncope units. Compared to the investigation of patients with syncope in the general cardiology department, both the diagnostic yield (57.9%) and the ECG-based diagnostic yield (18.3%) were improved in the syncope unit era, to 74.5% and 42.9%, respectively. Our data are in line with the findings of various other studies performed in different medical systems, indicating the great importance of the specialized, structured, and stepwise diagnostic approach used in this patient group [4,5,6,7,8,9,12,20,21]. ILR provided diagnosis in 72 cases, including 5 cases of reflex syncope with cardioinhibition and asystole, 17 cases of cardiogenic syncope with tachycardia (fast atrial fibrillation in 6 cases, supraventricular tachycardia in 8 cases, and ventricular tachycardia in 3 cases), and 50 cases of cardiogenic syncope with bradycardia (atrioventricular block in 31 cases and sinus block in 19 patients). In patients without diagnosis, the monitoring period was extended up to 3 years, whereas in those in whom a final diagnosis was achieved, the device was extracted 6 months after the time of diagnosis.

Despite the widely recognized importance of ILRs, these devices are prone to a variety of false-positive activations. To address this false-positive alert burden, manufacturers have recently implemented deep learning algorithms developed by using artificial intelligence (AI). Deep learning AI algorithms have been shown to improve device specificity without affecting accuracy. In addition, these ILRs are equipped with remote reprogramming ability, which, combined with customized initial programming, allows for adjustments in order to reduce false-positive events without requiring in-person visits. Thus, clinician time and costs from manually reviewing electrograms of false-positive alerts are further reduced.

This study has several limitations. Firstly, this was a retrospective, one-center study including a limited number of patients. Moreover, our patients were referred from various medical structures (e.g., primary care, other cardiology departments, other specialties), resulting in significant inhomogeneity at the depth of investigation prior to our assessment. Some of the patients had undergone an extensive investigation, whereas others were less well investigated. Third, the number of tests performed prior to our assessment was not recorded in detail, although some of them, such as 24 h ambulatory ECG monitoring, blood tests, and echocardiography, were not repeated if already performed. Fourth, based on our study design, we could not conclusively determine whether ILR implantation directly affects ECG diagnosis and the timing of diagnosis, independent of patient and syncope characteristics. Moreover, this study was conducted at a single center in Greece, which may limit the generalizability of the findings to other populations or settings. Lack of data from multiple centers or diverse populations makes it difficult to generalize the findings to other settings. In addition, a detailed cost analysis comparing the financial implications of different diagnostic strategies was not performed. Furthermore, in the present study, no direct comparison was performed with a standard care group not evaluated in the syncope unit, even though patients in Group C were treated prior to the establishment of the syncope unit in our hospital. Finally, although the decision to implant an ILR was based on the current scoring system proposed by the ESC, as well as on clinical experience suggesting arrhythmic syncope, more strict criteria for patient selection should have been adopted. A future prospective, multicenter study including data from a conventional treatment group directly compared with the group investigated in the syncope unit would be beneficial in shedding light on the identification of the most adequate pathway of investigation for unexplained syncope. Future studies should also more strictly define the criteria for ILR selection.

## 5. Conclusions

Dedicated syncope units can increase the diagnostic yield of syncope with unknown etiology. A selective use of ILRs according to the clinical and electrocardiographic characteristics represents an acceptable diagnostic pathway that increases the effectiveness of the structured SU approach, shortening the time to the final diagnosis and further preserving financial resources.

## Figures and Tables

**Table 1:** Patient demographics and clinical characteristics.

Characteristics	Group A SU + ILR (*n* = 133)	Group B SU-ILR (*n* = 63)	Group C No SU/No ILR (*n* = 71)	*p*-Value
Female, *n* (%)	66 (49.6%)	31 (49.2%)	37 (52.1%)	0.93
Age, years	67.3 ± 16.9	48.3 ± 19.1 *	57.8 ± 16.4 *†	<0.001
Characteristics of syncope/pre-syncope				
Syncope ± pre-syncope	122 (91.7%)	57 (90.5%)	66 (93%)	0.71
Pre-syncope only	11 (8.3%)	6 (9.5%)	5 (7%)	0.27
Traumatic syncope, *n* (%)	97 (72.9%)	39 (61.9%)	46 (64.8%)	0.23
Νumber of episodes	8.2 ± 2.1	3.8 ± 1.4 *	4.3 ± 1.5 *†	<0.001
Prodromes ^1^, *n* (%)	92 (69.2%)	57 (90.5%)	53 (74.6%)	0.05
Predisposing factors ^2^	23 (17.3%)	40 (63.5%)	37 (52.1%)	<0.001
Position, *n* (%) Supine Sitting Standing Changing Driving	12 (9%) 64 (48.1%) 93 (69.9%) 13 (9.8%) 3 (2.3%)	4 (6.3%) 28 (44.4%) 44 (69.8%) 13 (20.6%) 0 (0%)	-	0.80
Exertion status, *n* (%) Rest Mild Intense Post-exertion	85 (63.9%) 67 (50.4%) 4 (3%) 2 (1.5%)	41 (65.1%) 29 (46%) 3 (4.8%) 5 (7.9%)	-	0.14
Pre-syncope, *n* (%)	82 (61.7%)	30 (47.6%)	-	0.06
Palpitations, *n* (%)	22 (16.5%)	5 (7.9%)	-	0.10
Post syncope symptoms, *n* (%)	105 (78.9%)	41 (65%)	-	0.04
Mean recovery time (min)	5.2 ± 4.8	3.2 ± 4.1	-	<0.001
Clinical characteristics				
Arterial hypertension, *n* (%)	37 (27.8%)	12 (19%)	20 (28.2%)	0.22
Dyslipidemia, *n* (%)	33 (24.8%)	12 (19%)	23 (32.4%)	0.22
Diabetes mellitus, *n* (%)	17 (12.8%)	6 (9.5%)	7 (9.9%)	0.72
Smoking, *n* (%)	33 (24.8%)	19 (30.2%)	16 (22.5%)	0.58
Coronary heart disease, *n* (%)	19 (14.3%)	4 (6.3%)	8 (11.3%)	0.26
Other cardiomyopathy, *n* (%)	1 (0.8%)	0 (0%)	1 (1.4%)	0.83
Channelopathy, *n* (%)	2 (1.5%)	0 (0%)	0 (0%)	0.99
Atrial fibrillation, *n* (%)	21 (15.8%)	3 (4.7%)	8 (11.3%)	0.08
Pacemaker, *n* (%)	3 (2.6%)	2 (3.2%)	0	0.78
Valvular heart disease, *n* (%)	3 (2.6%)	0 (0%)	4 (5.6%)	0.30
Mitral valve prolapse, *n* (%)	3 (2.6%)	0 (0%)	0 (0%)	0.88
AVNRT, *n* (%)	2 (1.5%)	0 (0%)	0 (0%)	0.99
Operated ASD, *n* (%)	1 (0.8%)	0 (0%)	0 (0%)	0.88
Previous stroke, *n* (%)	2 (1.5%)	0 (0%)	2 (2.8%)	0.79
12-Lead abnormalities				
Sinus bradycardia, *n* (%)	23 (17.3%)	3 (4.8%)	5 (7%)	0.02
First-degree AV block, *n* (%)	5 (3.8%)	2 (3.2%)	3 (4.2%)	0.95
Bifascicular block, *n* (%)	7 (5.3%)	1 (1.6%)	1 (1.4%)	0.23
Atrial fibrillation, *n* (%)	10 (7.5%)	1 (1.6%)	3 (4.2%)	0.06
Left anterior fascicular block, *n* (%)	3 (2.3%)	1 (1.6%)	0 (0%)	0.89
Right bundle branch block, *n* (%)	7 (5.3%)	1 (1.6%)	2 (2.8%)	0.39
Left bundle branch block, *n* (%)	6 (4.5%)	2 (3.2%)	2 (2.8%)	0.08
Incomplete right bundle branch block, *n* (%)	0 (0%)	6 (9.5%)	1 (1.4%)	0.002
Left ventricular hypertrophy, *n* (%)	11 (8.3%)	6 (9.5%)	-	0.77
Ischemic changes, *n* (%)	1 (0.8%)	0 (0%)	0 (0%)	0.85
T-wave inversion, *n* (%)	11 (8.3%)	0 (0%)	3 (4.2%)	0.13
Prolonged QT, *n* (%)	1 (0.8%)	0 (0%)	0 (0%)	0.84
Brugada-type ECG changes, *n* (%)	2 (1.5%)	0 (0%)	0 (0%)	0.99
Early repolarization, *n* (%)	2 (1.5%)	3 (4.8%)	1 (1.4%)	0.30
Premature atrial contractions, *n* (%)	11 (8.3%)	5 (7.9%)	4 (5.6%)	0.78
Premature ventricular contractions, *n* (%)	3 (2.3%)	1 (1.6%)	4 (5.6%)	0.30
Paced, *n* (%)	2 (1.5%)	0 (0%)	0 (0%)	0.99
Low EGSYS score (<3%), *n* (%)	58 (43.6%)	27 (42.9%)	-	0.92

Note: * *p* < 0.05 compared to group A; † *p* < 0.05 compared to group B. ^1^ Prodromes included symptoms such as dizziness, nausea, sweating, abdominal pain, shortness of breath, palpitations, difficulty in maintaining body stature, tinnitus, etc., or a combination of the above symptoms. ^2^ Predisposing factors included situations such as psychological stress, acute pain, longstanding post-prandial period, post-urination/defecation period, standing, or a hot or crowded environment.

**Table 2:** Additional tests performed after initial evaluation with history, clinical examination, and ECG.

Test Performed	Group A SU + ILR (*n* = 133)	Group B SU-ILR (*n* = 63)	Group C No SU/No ILR (*n* = 71)	*p*-Value
Carotid massage, *n* (%)	32 (24.1%)	8 (12.7%)	-	0.03
Active standing, *n* (%)	133 (100%)	63 (100%)	-	1.0
Hematology/biochemistry, *n* (%)	133 (100%)	63 (100%)	-	1.0
Echocardiogram, *n* (%)	133 (100%)	63 (100%)	60 (84.6%)	0.001
Left ventricular ejection fraction	55 ± 10%	54 ± 8%	-	0.5
ECG monitoring, *n* (%)	18 (13.5%)	0 (0%)	-	0.002
Ambulatory 24 h ECG recording, *n* (%)	133 (100%)	63 (100%)	56 (78.9%)	0.001
Treadmill ECG stress test, SPECT, or stress echo test, *n* (%)	21 (15.8%)	11 (17.5%)	22 (31%)	0.03
Coronary catheterization, *n* (%)	6 (4.5%)	2 (3.2%)	11 (15.5%)	0.005
Tilt table test, *n* (%)	14 (10.5%)	50 (79.4%)	24 (33.8%)	<0.001
Electrophysiological study, *n* (%)	20 (15%)	4 (6.3%)	13 (18.4%)	0.11
Central nervous system computed tomography and/or magnetic resonance imaging, *n* (%)	2 (1.5%)	1 (1.6%)	4 (5.6%)	0.18

**Table 3:** Diagnostic yield and time to diagnosis.

	Group A SU + ILR (*n* = 133)	Group B SU-ILR (*n* = 63)	Group C No SU/No ILR (*n* = 71)	*p*-Value
Mean follow-up time, m	25.6 ± 10.6	28.1 ± 12.4	No systematic Follow-up	0.15
Established final diagnosis, *n* (%)	98 (73.7%)	48 (76.2%) *	38 (53.5%) *†	0.22
**Cause of syncope, *n* (%)**				
Reflex *Cardioinhibitory*	**23 (23.5%)** *5* (*21.7%*) ^1^	**37 (77.1%)** *6* (*16.2%*) ^1^	**22 (57.9%)** *n/a*	<0.001
Cardiogenic *Bradycardia* *Tachycardia* *AF* *SVT* *VT*	**68 (69.4%)** *50* (*73.5%*) ^2^ *6* (*8.8%*) ^2^ *8* (*11.8%*) ^2^ *3* (*4.4%*) ^2^	**6 (12.5%)** *3* (*50%*) ^2^ *0* (*0%*) ^2^ *1* (*16.7%*) ^2^ *2* (*33.3%*) ^2^	**13 (34.2%)** *7* (*53.8%*) ^2^ *2* (*15.4%*) ^2^ *3* (*23.1%*) ^2^ *1* (*7.7%*) ^2^	
Pump failure **Orthostatic hypotension**	*1* (*1.5%*) ^2^ **1 (1.0%)**	*0* (*0%*) ^2^ **3 (6.3%)**	*0* (*0%*) ^2^ **3 (7.9%)**	
Other (Non-syncopal) ^3^	**6 (6.1%)**	**2 (4.1%)**	**0 (0%)**	
Established ECG-based diagnosis, *n* (%)	72 (55.1%)	12 (19%)	13 (18.3%)	<0.001
Time to final diagnosis, months	4.2 ± 2.7	7.5 ± 5.6	-	<0.001

AF: atrial fibrillation; SVT: supraventricular tachycardia; VT: ventricular tachycardia. ^1^ Calculated as a proportion of reflex syncope. ^2^ Calculated as a proportion of cardiogenic syncope. ^3^ Other causes included 2 patients with drops, 1 patient with meningioma, 1 patient with epilepsy, 1 patient with persistent hyponatremia and hypokalemia, and 1 patient with pulmonary embolism in Group A, as well as 1 patient with psychogenic pseudo-syncope and 1 patient with hypoglycemia due to insulinoma in Group B; * *p* < 0.05 compared to Group A; † *p* < 0.05 compared to Group B. *n/a* Data of the nature of reflex syncope were not available.

## Data Availability

The original contributions presented in the study are included in the article, further inquiries can be directed to the corresponding author.
